# Supplementary material for: Predicting visual function by interpreting a neuronal wiring diagram
Source: Nature. 2024 Oct 2;634(8032):113–23. doi: 10.1038/s41586-024-07953-5 (PMC11446822; doi:10.1038/s41586-024-07953-5)
Supplement: Supplementary file 6 — CRF and ERF predictions for individual TmY4 and TmY9 cells. Analogous to Supplementary Data 3, but for TmY target types. Shown are the top four monosynaptic pathways, the strongest pathway passing through each of the top ten intermediary types (ranking from Extended Data Fig. 7), and the trisynaptic pathway Tm1–TmY–Dm3–TmY (see the section entitled Prediction of spatial normalization). [file 41586_2024_7953_MOESM6_ESM.zip › DataS4/TmY9q/720575940631262226.pdf]

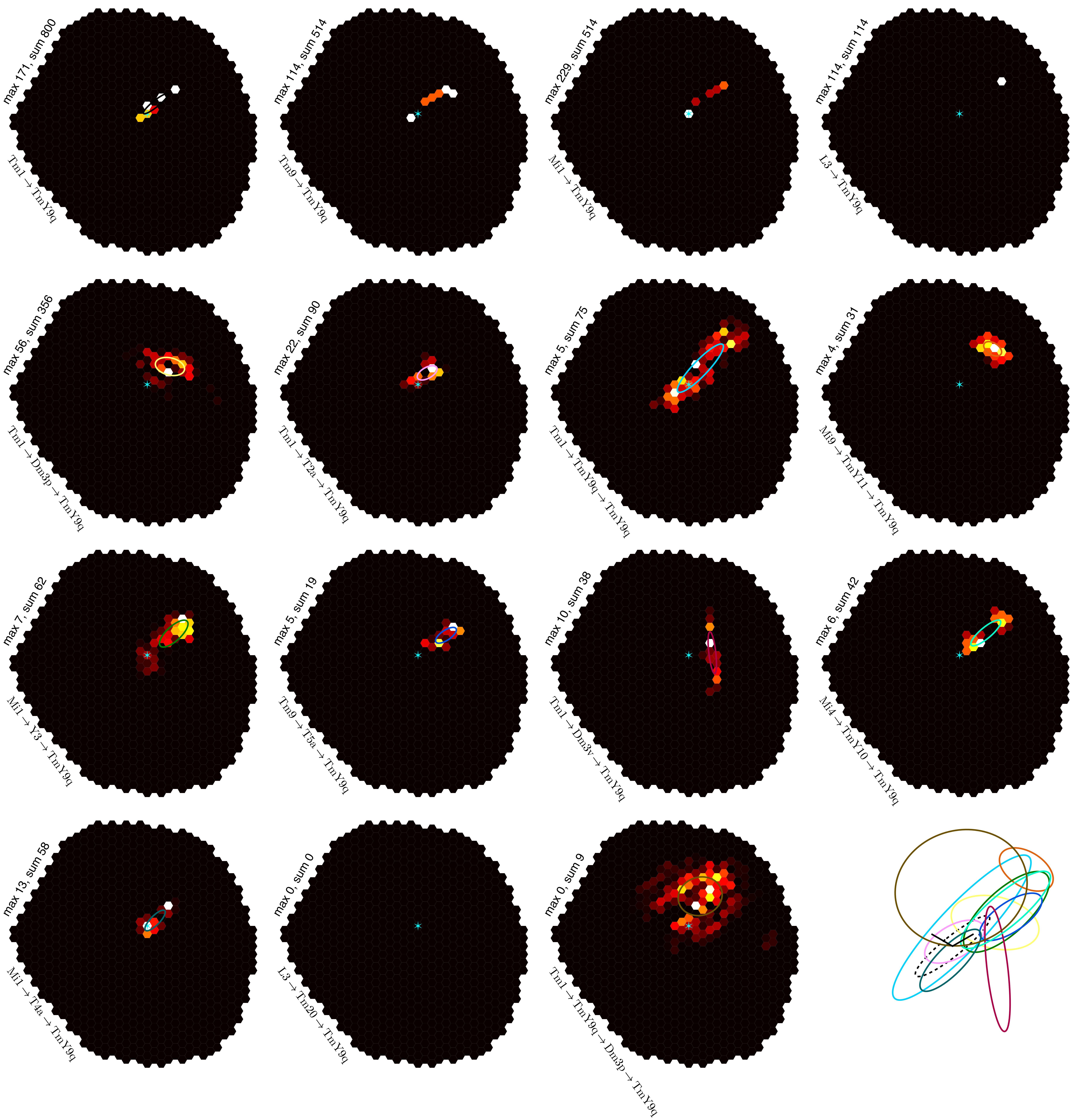

max 171, sum 800
Tm1 → TmY9q
max 114, sum 514
Tm9 → TmY9q
max 229, sum 514
Mi1 → TmY9q
max 114, sum 114
L3 → TmY9q
max 56, sum 356
Tm1 → Dm3p → TmY9q
max 22, sum 90
Tm1 → T2a → TmY9q
max 5, sum 75
Tm1 → TmY9q → TmY9q
max 4, sum 31
Mi9 → TmY11 → TmY9q
max 7, sum 62
Mi1 → Y3 → TmY9q
max 5, sum 19
Tm9 → T5a → TmY9q
max 10, sum 38
Tm1 → Dm3v → TmY9q
max 6, sum 42
Mi4 → TmY10 → TmY9q
max 13, sum 58
Mi1 → T4a → TmY9q
max 0, sum 0
L3 → Tm20 → TmY9q
max 0, sum 9
Tm1 → TmY9q → Dm3p → TmY9q
